# The Conceptualisation and Measurement of DSM-5 Internet Gaming Disorder: The Development of the IGD-20 Test

**DOI:** 10.1371/journal.pone.0110137

**Published:** 2014-10-14

**Authors:** Halley M. Pontes, Orsolya Király, Zsolt Demetrovics, Mark D. Griffiths

**Affiliations:** 1 Nottingham Trent University, Psychology Division, Nottingham, United Kingdom; 2 International Gaming Research Unit, Nottingham Trent University, Nottingham, United Kingdom; 3 Departament of Clinical Psychology and Addiction, Institute of Psychology, Eötvös Loránd University, Budapest, Hungary; University of Florida, United States of America

## Abstract

**Background:**

Over the last decade, there has been growing concern about ‘gaming addiction’ and its widely documented detrimental impacts on a minority of individuals that play excessively. The latest (fifth) edition of the American Psychiatric Association's *Diagnostic and Statistical Manual of Mental Disorders* (DSM-5) included nine criteria for the potential diagnosis of Internet Gaming Disorder (IGD) and noted that it was a condition that warranted further empirical study. Aim: The main aim of this study was to develop a valid and reliable standardised psychometrically robust tool in addition to providing empirically supported cut-off points.

**Methods:**

A sample of 1003 gamers (85.2% males; mean age 26 years) from 57 different countries were recruited via online gaming forums. Validity was assessed by confirmatory factor analysis (CFA), criterion-related validity, and concurrent validity. Latent profile analysis was also carried to distinguish disordered gamers from non-disordered gamers. Sensitivity and specificity analyses were performed to determine an empirical cut-off for the test.

**Results:**

The CFA confirmed the viability of IGD-20 Test with a six-factor structure (salience, mood modification, tolerance, withdrawal, conflict and relapse) for the assessment of IGD according to the nine criteria from DSM-5. The IGD-20 Test proved to be valid and reliable. According to the latent profile analysis, 5.3% of the total participants were classed as disordered gamers. Additionally, an optimal empirical cut-off of 71 points (out of 100) seemed to be adequate according to the sensitivity and specificity analyses carried.

**Conclusions:**

The present findings support the viability of the IGD-20 Test as an adequate standardised psychometrically robust tool for assessing internet gaming disorder. Consequently, the new instrument represents the first step towards unification and consensus in the field of gaming studies.

## Introduction

Over the last decade, there has been growing worldwide concern from researchers about ‘gaming addiction’. Official bodies such as the American Psychiatric Association [1] and numerous scholars [2]–[5] have suggested the need for unification and consensus for the assessment of gaming addiction if this phenomenon is to be considered as an independent clinical entity in the future. Despite the proliferation of research on gaming behaviour over the last few years [6], [7], the field has been hindered by the use of inconsistent and non-standardised criteria to assess and identify problematic and/or addictive video game use [6]. Moreover, this problem may be also reflected by the heterogeneity of nomenclatures used by researchers to address the same phenomenon including such terms as video game addiction [8], computer game playing dependence [9], internet addiction disorder [10], video game dependency [11], problematic online gaming [12], and pathological video-game use [13]. In addition to these issues, most psychometric tools developed for assessing behavioural addictions (including gaming addiction) have either used an *ad hoc* cut-off point or lacked a strong empirical base for establishing such cut-off points.

These problems may be partially explained by the lack of agreement amongst researchers on how to approach the assessment of the phenomenon. For instance, some studies [13], [14] adapted the definition of pathological gambling from the *Diagnostic and Statistical Manual of Mental Disorders* (4th ed.; DSM-IV; [15]) to assess this phenomenon. Others have been based on the DSM-IV criteria of substance use dependence [16], or have combined these two approaches and used criteria from both pathological gambling and substance use dependence [17]. Additionally, some researchers have used criteria from various different behavioural addictions such as internet addiction [18] or exercise addiction [19].

In acknowledgement of the many studies now published in the area of problematic gaming, Section 3 of the fifth revision of the DSM [1] included ‘internet gaming disorder’ (IGD) for the first time. Here, IGD was viewed as a behavioural addiction that needs further study before being recognised as an independent clinical disorder. This represents a milestone achievement by attempting to (i) provide a consensual view of the phenomenon from a scientific point of view, and (ii) unify different approaches into a single one [3].

According to the APA [1], the clinical diagnosis of IGD comprises a behavioural pattern encompassing persistent and recurrent use of the Internet to engage in games, leading to significant impairment or distress in a period of 12 months as indicated by five (or more) out of the nine criteria that must be present. More specifically, the nine proposed criteria for IGD include: (1) preoccupation with internet games; (2) withdrawal symptoms when internet gaming is taken away; (3) tolerance, resulting in the need to spend increasing amounts of time engaged in internet games; (4) unsuccessful attempts to control participation in internet games; (5) loss of interests in previous hobbies and entertainment as a result of, and with the exception of, internet games; (6) continued excessive use of internet games despite knowledge of psychosocial problems; (7) deceiving family members, therapists, or others regarding the amount of internet gaming; (8) use of internet games to escape or relieve negative moods; and (9) jeopardising or losing a significant relationship, job, or education or career opportunity because of participation in internet games. Furthermore, it has been asserted that IGD may lead to school/college failure, job loss, or marriage failure as the compulsive gaming behaviour tends to displace usual and expected social, work and/or educational, relationship, and family activities [1]. It has also been noted [3] that the nine IGD criteria directly map onto the six criteria of Griffiths' components model of addiction (i.e., salience, mood modification, tolerance, withdrawal symptoms, conflict and relapse) [20].

The aim of the present study was twofold. Our main goal was to examine whether the nine IGD criteria from the DSM-5 [1] can empirically correspond with the six dimensions of the components model of addiction by developing a new standardised psychometric tool. Our second goal was to provide evidence of its reliability and validity alongside an empirical cut-off point for future studies wishing to assess IGD in line with the DSM-5. If the results of the study support these two aims, then the newly developed tool represents a valuable instrument for future researchers to empirically investigate IGD.

## Methods

This study was approved by the College Research Ethics Committee of Nottingham Trent University (UK). In order to participate in the study informed consent was sought amongst participants and the minimum age of participation in the study was 16 years old.

### Sample, Procedure, and Participants

Participants were invited to take part in the study by clicking the survey link provided in 52 online gaming forums. In order to advertise the survey a thread was created and daily checked for a month on each of the 52 online forums specifying the nature of the study. The survey was created and hosted online. The online data collection methodology was chosen because of its inherent benefits, such as ease of access to larger sample pools, cost-efficiency, and its usefulness and practical advantages for researching behavioural addictions in general [21], [22], especially in the case of online gamers. This methodology might also increase participant's self-disclosure [23] and disinhibition [24], which helps to decrease social desirability. A total of 1397 questionnaires were collected. However, 394 of these (28.2%) were not fully completed and were therefore excluded from the subsequent analyses.

### Measures

#### Socio-demographics

Information regarding gender, age, country of residence, age when they first began gaming, relationship status, ownership of mobile device with internet access and/or gaming console and other gaming devices were collected.

#### Weekly Gameplay

This variable examined the player's weekly time spent gaming on computers, consoles, and/or other gaming platforms (e.g., handheld devices). This was operationalised into distinct playing categories (i.e., less than 7 hours a week; between 8 and 14 hours a week; between 15 and 20 hours a week; between 21 and 30 hours a week; between 31 and 40 hours a week, and more than 40 hours per week). This variable was later recoded to distinguish between players that played more or less than 30 hours a week in order to fully reflect APA's definition of IGD concerning the time spent playing.

#### Internet Gaming Disorder Test

The IGD-20 Test includes 20 items reflecting the nine criteria of IGD as in the DSM-5 [1] and incorporated the theoretical framework of the components model of addiction (i.e., salience, mood modification, tolerance, withdrawal symptoms, conflict and relapse) [20]. Consequently, three items were devised for each of the following IGD criteria 1, 2, 3, 4 and 8 and another five items for criteria 5, 6, 7 and 9 altogether because these latter four criteria appear to reflect the conflict dimension (see Table 1). The IGD-20 Test examines both online and/or offline gaming activities occurring over a 12-month period, since the DSM-5 criteria for IGD are based on persistent and recurrent gaming. This most often involves specific internet games, but can also include non-internet computerised games [1]. Participants rated all items of this test on a 5-point Likert scale: 1 (“Strongly disagree”), 2 (“Disagree”), 3 (“Neither agree or disagree”), 4 (“Agree”), and 5 (“Strongly agree”).

**Table 1:** Model Comparison: “Components” Model (Griffiths, 2005) vs. Internet Gaming Disorder DSM-5 nine criteria (APA, 2013).

Components Model (Griffiths, 2005)	Internet Gaming Disorder DSM-5 (APA, 2013)	
Salience	**1**	1. Preoccupation with Internet Games (The individual thinks about previous gaming activity or anticipates playing the next game; Internet gaming becomes the dominant activity in daily life.
Mood Modification	**8**	8. Use of Internet Games to escape or relieve a negative mood (e.g., feelings of helplessness, guilt, anxiety).
Tolerance	**3**	3. Tolerance – the need to spend increasing amounts of time engaged in Internet games.
Withdrawal	**2**	2. Withdrawal Symptoms when Internet gaming is taken away. (These symptoms are typically described as irritability, anxiety, or sadness, but are no physical signs of pharmacological withdrawal.
Conflict	**5, 6, 7 and 9**	5. Loss of interests in previous hobbies and entertainment as a result of, and with the exception of, Internet games.
		6. Continued excessive use of Internet games despite knowledge of psychosocial problems.
		7. Has deceived family members, therapists, or others regarding the amount of Internet gaming.
		9. Has jeopardised or lost a significant relationship, job, or educational career opportunity because of participation in Internet games.
Relapse	**4**	4. Unsuccessful attempts to control the participation in Internet games.

#### Diagnostic Criteria of IGD in DSM-5

The diagnostic features of the IGD in DSM-5 comprise nine criteria reflecting its key aspects. According to the APA [1], to be diagnosed with IGD a person has to endorse at least five (or more) of the nine criteria over a 12-month period. Since these nine criteria were developed to be used by clinicians as a form of checklist in a binary system (i.e., yes or no), we slightly modified the response option so that it could be presented to participants along a continuum using a 5-point scale (i.e., 1 “Never”, 2 “Rarely”, 3 “Sometimes”, 4 “Often”, 5 “Very Often”). This was done because the research team felt the restrictive two-option (yes/no) choice might be problematic from a statistical standpoint [25]. Additionally, previous research suggested that multiple-choice items traditionally yield more reliable test scores than scores derived from dichotomous items [26]. In the present study, the Diagnostic Criteria of IGD's internal consistency as measured by the Cronbach's alpha was.87.

### Statistical Analysis

In order to test the proposed model for IGD, confirmatory factor analysis (CFA) was performed with maximum likelihood estimation with robust standard errors (MLR) in MPLUS 6.1 [27]. The goodness of fit was evaluated using a *p* value of Chi-square smaller than.05 for the test of close fit. Additional fit indices included the comparative fit indices (CFI), Tucker-Lewis Fit index (TLI), root mean square error of approximation (RMSEA) and its 90% confidence interval (90% CI), and standardised root mean square residual (SRMR). A model presents an acceptable fit by a CFI greater than.90 and a RMSEA value smaller than.08. A good fit is expressed by a CFI value higher than.95 and a RMSEA value close to.06 [28], [29].

In order to identify the groups of gamers with higher risk of IGD, latent profile analysis (LPA) was performed in MPLUS 6.1 [27]. The LPA is a mixture modeling technique used to identify groups of people that are similar in their responses to certain variables – in this case average sum scores given for the six IGD-20 Test dimensions (continuous manifest variables) [30]. In the process of determining the number of latent classes, the Bayesian information criteria parsimony index was used, alongside the minimisation of cross-classification probabilities, entropy and the interpretability of clusters. In the final determination of the number of classes, the likelihood-ratio difference test (Lo-Mendell-Rubin Adjusted LRT Test) was also used. This compares the estimated model with a model having one less class than the estimated model [27]. A low *p* value (<.05) suggests that the model with one less class is rejected in favour of the estimated model.

To determine the cut-off points of the IGD-20 Test, a sensitivity analysis based on membership in the “disordered gamers” group from the latent profile analysis as the ‘gold standard’ was carried out. Thus, the accuracy of the IGD-20 Test by calculating the proportion of participants classified as ‘disordered gamers’ versus other gamers could be assessed. The sensitivity (i.e., the proportion of true positives belonging to the disordered group based on LPA) and specificity (i.e., the proportion of true negatives among the non-disordered gamers) were defined as suggested by Altman and Bland [31] and Glaros and Kline [32]. In order to explore the probability that the IGD-20 Test would give the correct ‘diagnosis’, the positive predictive values (PPVs), the negative predictive values (NPVs), and the accuracy values for each possible IGD-20 Test cut-off points were calculated. PPV was defined as the proportion of participants with positive test results who are correctly diagnosed [32], [33]. The NPV was defined as the proportion of participants with negative test results who are correctly diagnosed [32], [33].

Additionally, to assess the validity of the IGD-20 Test, the LPA classes were compared alongside other variables (i.e., gender, age, weekly gameplay, IGD-9 scores, and IGD-20 Test scores) relevant to the phenomenon of IGD. In order to do these comparisons, Wald's Chi-square test of mean equality for latent class predictors in mixture modeling was also performed because it takes into account the probabilistic nature of the LPA groups (for description of analysis, see www.statmodel.com/download/meantest2.pdf).

## Results

### Descriptive Statistics

The total sample comprised 1003 participants, with the majority (85.2%) being male (n = 855). Ages varied between 16 and 58 years, and the mean age was 26 years (SD = 8.2 years). All sample characteristics are presented in Table 2.

**Table 2:** Socio-Demographic Characteristics of the Sample.

N	1003
Gender (male, n, %)	85.2
Age, years; Mean (SD)	26.5 (0.26)
Country (n, %)	
United Kingdom	281 (28)
United States	212 (21.1)
Sweden	66 (6.6)
Netherlands	48 (4.8)
Germany	38 (3.8)
Canada	34 (3.4)
Finland	31 (3.1)
Other countries	293 (29.2)
Weekly Gameplay (n, %)	
More than 30 hours	260 (25.9)
Relationship Status (n, %)	
In a relationship	450 (44.9)
Use of Substance> 3 times a week (n, %)	
Cigarettes	155 (15.5)
Alcohol	113 (11.3)
Owning a mobile phone with Internet access (n, %)	862 (85.9)
Owning a game console or other dedicated gaming device (n, %)	708 (70.6)

### Confirmatory Factor Analysis

The analysis of the first-order model with the six factors (i.e., salience, mood modification, tolerance, withdrawal symptoms, conflict, and relapse) provided an acceptable model fit for the IGD-20 Test, χ^2^ (151, n = 1003) = 504.6, *p*<0.0001; CFI = 0.935; TLI = 0.918 RMSEA = 0.048 (90%CI: 0.044-0.053), *p*close = 0.716; SRMR = 0.041 (see Table 3). With the exception of item 19, all factor loadings were higher than.50 with their respective factors. The correlations among the factors ranged from.42 to.94, with the highest correlation observed being between salience and tolerance and the lowest between mood modification and conflict (see Table 4).

**Table 3:** Confirmatory Factor Analysis of the 20 items of the IGD-20 Test.

	Salience	Mood Modification	Tolerance	Withdrawal Symptoms	Conflict	Relapse
1. I often lose sleep because of long gaming sessions.	.61					
7. I usually think about my next gaming session when I am not playing.	.57					
13. I think gaming has become the most time consuming activity in my life.	.67					
8. I play games to help me cope with any bad feelings I might have.		.87				
2R. I never play games in order to feel better.		.60				
14. I play games to forget about whatever's bothering me.		.76				
3. I have significantly increased the amount of time I play games over last year.			.56			
9. I need to spend increasing amounts of time engaged in playing games.			.64			
15. I often think that a whole day is not enough to do everything I need to do in-game.			.59			
4. When I am not gaming I feel more irritable.				.75		
10. I feel sad if I am not able to play games.				.71		
16. I tend to get anxious if I can't play games for any reason.				.82		
5. I have lost interest in other hobbies because of my gaming.					.59	
11. I have lied to my family members because the amount of gaming I do.					.65	
19R. I know my main daily activity (i.e., occupation, education, homemaker, etc.) has not been negatively affected by my gaming.					.47	
17. I think my gaming has jeopardised the relationship with my partner.					.52	
20. I believe my gaming is negatively impacting on important areas of my life.					.70	
6. I would like to cut down my gaming time but it's difficult to do.						.61
12. I do not think I could stop gaming.						.50
18. I often try to play games less but find I cannot.						.66

Empty cells represents the factor loadings that are fixed to 0; all other factor loadings are significant at least at p<.001. Cronbach's alpha of the total 20 items of the Internet Gaming Disorder Test is.88.

**Table 4:** Summary of the Confirmatory Factor Analysis Results on the IGD-20 Test Items.

Salience	1	.47	.94	.70	.74	.69
Mood Modification		1	.49	.45	.42	.48
Tolerance			1	.77	.66	.72
Withdrawal Symptoms				1	.63	.63
Conflict					1	.86
Factor determinacies	.90	.91	.90	.92	.89	.88
Cronbach's a	.64	.78	.63	.80	.74	.63
Mean	2.81	3.06	2.29	2.08	2.18	2.35
SD	.93	.98	.87	.88	.81	.83

### Criterion-related Validity, Concurrent Validity, and Reliability

Criterion-related validity was assessed by the association between weekly gameplay and the IGD-20 Test scores (*r_s_*(1003) = .77, *p*<.001). Although time spent on games itself should not be the sole indicator of IGD, disordered players typically devote between 8 to 10 hours or more per day to gaming activity and at least 30 hours per week [1]. Therefore, the strong correlation between these two variables was considered an evidence of criterion-related validity. Concurrent validity was assessed by the association of the IGD-20 Test with the nine IGD criteria from the DSM-5 (*r_s_* (1003) = .82, *p*<.001). Additionally, the six IGD-20 Test dimensions were strongly correlated with their corresponding IGD criteria (see Table 5). The IGD-20 Test's internal consistency as measured by the Cronbach's alpha was.88.

**Table 5:** Overall correlation between the six IGD-20 Test factors and its corresponding IGD nine criteria.

IGD DSM-5 nine Criteria	IGD Test (Six Factors)					
	Salience	Mood Modification	Tolerance	Withdrawal Symptoms	Conflict	Relapse
**IGD1**	**.58** **	.25**	.43**	.43**	.44**	.40**
**IGD2**	.45**	.36**	.42**	**.63** **	.48**	.44**
**IGD3**	.44**	.28**	**.49** **	.47**	.48**	.44**
**IGD4**	.40**	.30**	.42**	.45**	.54**	**.56** **
**IGD5**	.37**	.23**	.32**	.39**	**.61** **	.39**
**IGD6**	.43**	.28**	.34**	.40**	**.59** **	.41**
**IGD7**	.38**	.23**	.32**	.34**	**.60** **	.38**
**IGD8**	.32**	. **71** **	.31**	.37**	.32**	.34**
**IGD9**	.35**	.24**	.32**	.35**	**.57** **	.37**

** Correlation is significant at the.01 level (2-tailed).

*** For a more comprehensive review on how the 9 IGD criteria overlap with each one of the six factors outlined see Griffiths, M. D., King, D., Demetrovics, Z. (2014). DSM-5 internet gaming disorder needs a unified approach to assessment. Neuropsychiatry, 4(1), 1–4.

### Latent Profile Analysis

After performing the LPA on the six dimensions of the IGD Test, a five-class solution was found according to the adopted decision criteria. As shown in Table 6, the AIC, the BIC, and sample-size adjusted BIC continued to decrease as more latent classes were added. However, a levelling-off after the five-latent-class solution was observed. In inspection of entropy, the five-class solution provided an adequate level. Based on the L-M-R test, the five-class solution was accepted.

**Table 6:** Results Obtained from the Latent Profile Analysis.

Number of latent classes	AIC	BIC	SSABIC	Entropy	L-M-R test	*p*
2 classes	14315	14409	14349	0.775	1259	<0.001
3 classes	13912	14039	13957	0.777	410	0.034
4 classes	13775	13938	13833	0.752	147	0.003
**5 classes**	**13688**	**13884**	**13757**	**0.753**	**100**	**0.048**
6 classes	13660	13890	13741	0.762	41	0.407

**Figure 1:**
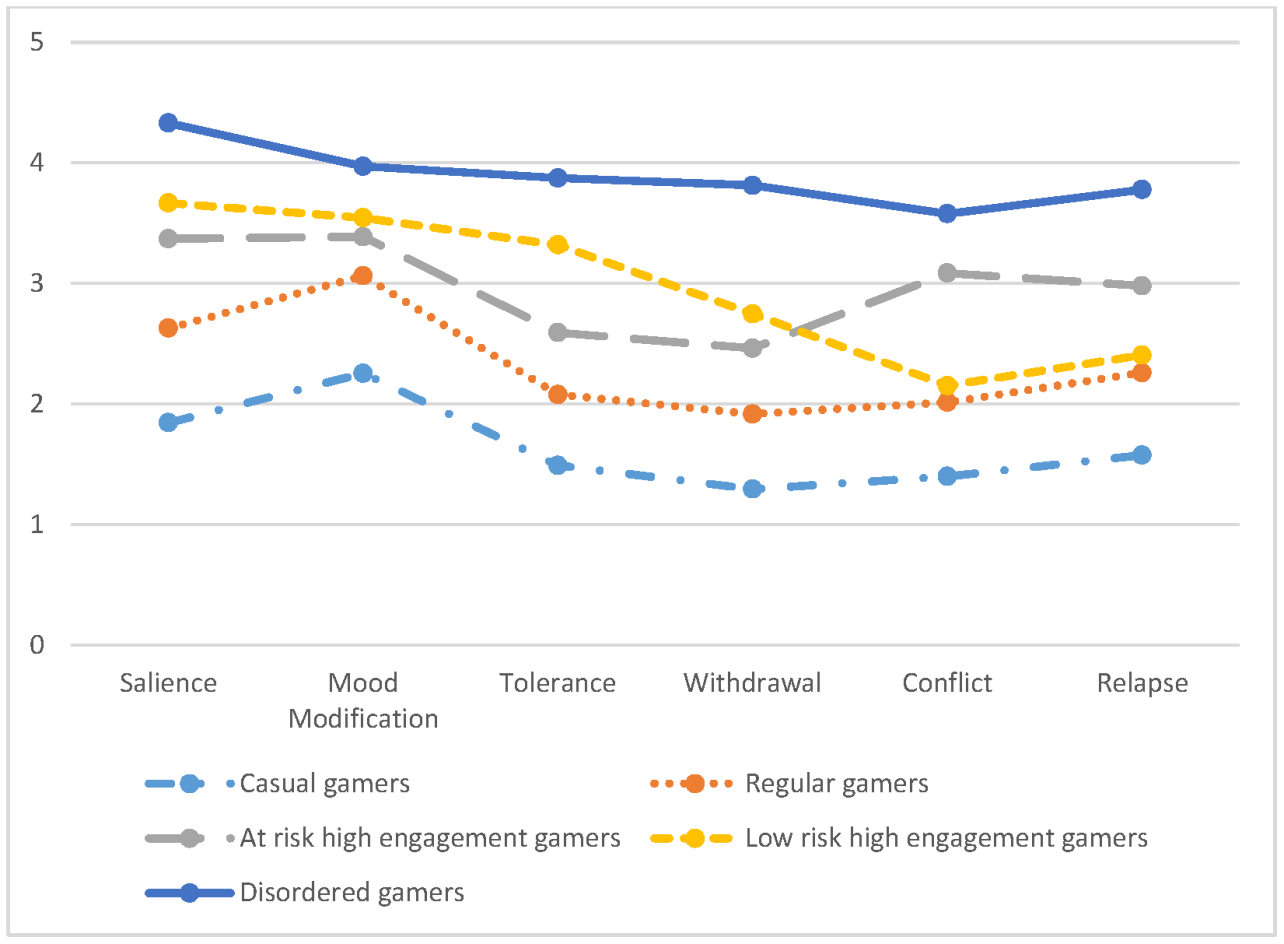
The Five Classes Obtained from the Latent Profile Analysis.

The features of the five classes are presented in Figure 1 and Table 7. The first and the second classes represent *casual gamers* (19.1%) and *regular gamers* (48.6%), that is, gamers that generally scored below the mean average. The third class represents *low risk engagement gamers* (10.4%), while the fourth class represents *at risk high engagement gamers* (16.7%). The main difference between these two classes is that the at risk high engagement gamers scored much higher on conflict and relapse, while the low risk high engagement gamers scored slightly higher in salience, mood modification, tolerance, and withdrawal symptoms. The final (fifth) class represents the *disordered gamers* (5.3%) that scored much higher on all six dimensions than the other four groups of gamers. Those players in the disordered gamers class were more likely to (i) be male, (ii) play for more than 30 hours per week, and (iii) have an overall higher score on the nine IGD criteria and IGD-20 Test (see Table 8).

**Table 7:** Comparison of the five latent classes: Testing Equality for Latent Class Predictors.

	Casual gamers (N = 192)	Regular gamers (N = 487)	Low risk high-engagement gamers (N = 104)	At risk high engagement gamers (N = 167)	Disordered gamers (N = 53)	Overall test	
						Wald χ^2^	*p value*
Salience, (min 1, max 5, mean 2.81 (SD = 0.93)), Mean (SE)	1.84 (0.05)_a_	2.64 (0.04)_b_	3.67 (0.08)_c_	3.38 (0.06)_d_	4.36 (0.10)_e_	286.5	<0.001
Mood Modification**, (min 1, max 5, mean 3.06 (SD = 0.98)), Mean (SE)	2.28 (0.07)_a_	3.06 (0.05)_b_	3.55 (0.11)_c_	3.38 (0.08)_c_	3.95 (0.12)_d_	91.5	<0.001
Tolerance, (min 1, max 5, mean 1.97 (SD = 0.74)), Mean (SE)	1.50 (0.05)_a_	2.09 (0.03)_b_	3.34 (008)_c_	2.60 (0.06)_d_	3.89 (0.12)_e_	184.6	<0.001
Withdrawal Symptoms, (min 1, max 5, mean 2.29 (SD = 0.87)), Mean (SE)	1.27 (0.04)_a_	1.90 (004)_b_	2.75 (0.09)_c_	2.46 (0.07)_d_	3.83 (0.11)_e_	243.9	<0.001
Conflict, (min 1, max 5, mean 2.18 (SD = 0.81)), Mean (SE)	1.40 (0.04)_a_	2.01 (0.03)_b_	2.14 (0.07)_b_	3.10 (0.06)_c_	3.60 (0.09)_d_	770.1	<0.001
Relapse, (min 1, max 5, mean 2.35 (SD = 0.83)), Mean (SE)	1.57 (0.05)_a_	2.25 (0.04)_b_	2.40 (0.08)_b_	3.00 (0.06)_c_	3.78 (0.13)_d_	364.9	<0.001

*Means having different subscript letters are different on at least p<.05 level according to the pairwise Wald *χ^2^* test of mean equality for latent class predictors in mixture modeling (http://bit.ly/NNCxju).

**Table 8:** Comparison of the five latent classes: Testing Equality for Latent Class Predictors.

	Casual gamers (N = 192)	Regular gamers (N = 487)	Low risk high-engagement gamers (N = 104)	At risk high engagement gamers (N = 167)	Disordered gamers (N = 53)	Overall test	
						Wald χ^2^	*p value*
Gender (Male %)	84.4_a_	85.5_a_	82.6_a_	87.2_a_	85.7_a_	1.2	0.875
Age (years), Mean (SE)	29.7 (0.77)_a_	27.0 (0.44)_b_	24.0 (0.83)_cd_	25.6 (0.70)_bd_	22.9 (0.97)_ec_	12.2	0.016
Weekly Gameplay (≥30 h) %	10.7 (0.02)_a_	20.7 (0.07)_b_	42.6 (0.16)_c_	34.0 (0.13)_c_	66.4 (0.17)_d_	31.4	<0.001
IGD9 (min 1, max 5, mean 1.97 (SD = 0.74)), Mean (SE)	1.23 (0.04)_a_	1.76 (0.03)_b_	2.14 (0.07)_c_	2.58 (0.06)_d_	3.58 (0.12)_e_	397.9	<0.001
IGD20 (min 1, max 5, mean 2.43 (SD = 0.64)), Mean (SE)	1.72 (0.03)_a_	2.27 (0.02)_b_	2.93 (0.04)_c_	3.01 (0.02)_c_	3.95 (0.06)_d_	1131.5	<0.001

*Means having different subscript letters are different on at least p<.05 level according to the pairwise Wald *χ^2^* test of mean equality for latent class predictors in mixture modeling (http://bit.ly/NNCxju).

### The empirical cut-off for determining the disordered gamers: Sensitivity and specificity analyses

As shown in Table 9, the sensitivity, specificity, positive predictive value (PPV), negative predictive value (NPV), and accuracy of the IGD-20 Test at possible cut-off points were calculated considering the membership in the fifth class (i.e., disordered gamers) as the ‘gold standard’. Based on this analysis, a cut-off score of 71 is suggested as an ideal empirical cut-off to distinguish disordered gamers from non-disordered gamers.

**Table 9:** Cut-Off Points for the IGD-20 Test based on the Fifth class (high addiction risk group) derived from the Latent Profile Analysis.

Cut-off	True positive	True negative	False positive	False negative	Sensitivity (%)	Specificity (%)	PPV (%)	NPV (%)	Accuracy (%)
66	53	912	38	0	100	96	58	100	96
67	53	920	30	0	100	97	64	100	97
68	51	930	20	2	96	98	72	100	98
69	51	942	8	2	96	99	86	100	99
70	51	945	8	2	96	99	86	100	99
**71**	**51**	**947**	**3**	**2**	**96**	**100**	**94**	**100**	**100**
72	48	950	0	5	91	100	100	99	100
73	43	950	0	10	81	100	100	99	99
74	41	950	0	12	77	100	100	99	99
75	34	950	0	19	64	100	100	98	98
6	28	950	0	25	53	100	100	97	98
77	24	950	0	29	45	100	100	97	97
78	21	950	0	32	40	100	100	97	97
79	19	950	0	34	36	100	100	97	97

In this case, the specificity is 100%, while the sensitivity is 96%. That is, practically none of the non-disordered cases are considered as disordered, while only 4% of the truly disordered gamers are not identified by the measure. Additionally, PPV is 94% and NPV is 100%. In other words, only 6% of the individuals with a positive test result are mistakenly identified, while all individuals with negative test results are identified correctly. The accuracy was 100%. Increasing the cut-off points would result in more false negative cases, while decreasing would increase the number of gamers mistakenly diagnosed.

## Discussion

Based on the need for a unified psychometrically sound measurement tool for the assessment of Internet Gaming Disorder (IGD), the present study aimed to develop and construct the IGD-20 Test based on a solid theoretical framework (i.e., components model of addiction) integrating in its model the nine IGD criteria presented in the DSM-5 as proposed by the American Psychiatric Association [1]. When administered to a large sample of heterogeneous gamers, the IGD-20 Test appeared to be an appropriate instrument for assessing IGD.

Overall, the psychometric analyses of the IGD-20 Test yielded good results in terms of validity and reliability. Additionally, the present model appears to have an acceptable model fit according to the results obtained from the CFA. More specifically, criterion-related and concurrent validity were warranted by the observed significant correlations between the (i) IGD-20 Test and weekly gameplay, and (ii) IGD-20 Test and the nine IGD criteria from DSM-5. Additionally, significant correlations between the IGD-20 Test's six factors and its corresponding IGD criteria also supported the test's concurrent validity. According to the latent profile analysis, 5.3% of the players belonged to the disordered gamers group, indicating a relatively conservative prevalence of disordered gamers among the sample, and is in line with other previously published and nationally representative studies (e.g., [11], [13]).

Previous research has attempted to distinguish between ‘addicted’ and ‘highly engaged’ players. Highly engaged players are non-disordered gamers displaying high levels of cognitive salience, tolerance and euphoria, while addicted players are those that display high levels of conflict, withdrawal, relapse, and behavioural salience in the first place [34], [35]. Interestingly, the *low risk high engagement gamers* group as shown in the LPA analysis, matched the profile described by Charlton and Danforth as highly engaged players. Hence, this group scored high on salience, mood modification, and tolerance, while scoring lower on the core components of addiction (conflict, withdrawal, and relapse). On the other hand the *at risk high engagement group* scored high on two core addiction components (conflict and relapse) in addition to scoring high on salience and mood modification. Although this group does not perfectly match the ‘addiction’ group defined by Charlton and Danforth [35], when compared to the *low risk high engagement* LPA group they might be at greater risk due to a higher displacement of conflict and relapse components. Therefore, in addition to using the suggested cut-off score (i.e., 71) to identify *disordered gamers*, we propose the use of a ‘pattern analysis’ for the remaining gamers to distinguish between low risk and at risk high engagement players. Players scoring high on the conflict, withdrawal, and relapse dimensions might be at greater risk than those scoring lower on these dimensions based on Charlton and Danforth's findings. The disordered gamers group was more likely to be male, and play for more than 30 hours per week. This finding is supported by other studies that found higher rates of addiction among males [11], [36]–[38], and those that found addicted gamers spend significantly more time playing than non-addicted players [13], [37], [39].

Finally, the sensitivity and specificity analysis revealed an empirically optimal cut-off of 71 points for diagnosing IGD with the IGD-20 Test. Nevertheless, future studies should further assess this in a clinical sample in order to corroborate the present findings. Recent research has already addressed this issue using the original nine criteria for IGD as a semi-structured interview [40]. However, this should also be done using a standardised and unified measurement tool in order to warrant progress and unification of the field.

The present study is not without limitations. The study used a convenience sample of gamers that was self-selecting (and therefore was not necessarily representative of all gamers). Consequently, the findings need to be cautiously interpreted in terms of generalizability. Notwithstanding, future studies should aim to confirm or disconfirm these results in representative samples (at either a national level and/or among the gaming community). Another important and difficult issue to overcome is the use of self-report questionnaires and their associated possible biases (e.g., social desirability biases, short-term recall biases, etc.). Future research should also attempt to confirm these findings using behavioural data and assess IGD in clinical samples in order to achieve recognition of this disorder as an independent clinical entity that merits inclusion in future editions of the DSM. Future studies could also include such measures as the Marlowe-Crowne Social Desirability Scale [41] to help overcome such biases (although this would lengthen such surveys and may lead to less participants completing them). This may also be related to the issue of non-completion of the survey. In the present study, just over 28% of the participants started but did not finish the survey. There is no way of knowing why the non-completion rate was so high, but this may be related to the survey being too long and/or gamers wanting to know what the survey was about with no intention of completing it (i.e., doing it out of curiosity). Whether the non-completers were any different from those gamers that completed the survey is not known, but this should be taken into account when considering the study's findings. Finally, participants in the present study were recruited from English-speaking online forums and communities, therefore, they were not filtered based on their first language. This may represent a possible limitation in that such people may not have fully understood the questions being asked. Therefore, future studies should take into account the first language of the participants.

Taken as a whole, the findings of the present study support the concept of IGD. It also supports the viability of its further study as reflected by the nine IGD criteria and the components model of addiction. Furthermore, the current findings also suggest that the IGD-20 Test satisfies the need for a standardised and psychometrically sound measurement tool for assessing this behavioural addiction in accordance to the IGD criteria outlined in DSM-5 [1]. Additionally, the IGD-20 Test was designed to be applicable and cover all gamers irrespective of the genre played, demarcating from previous trend of researching and assessing specific games and gamers such as those that play Massively Multiplayer Online Role Playing Games [6], [34]. Consequently, our hope is that this instrument facilitates the need to reach a consensus in the field in terms of assessment and conceptual definition of this increasingly studied phenomenon.

## Supporting Information

Table S1 **The Internet Gaming Disorder Test, Dimensionality and Instructions.**
(DOCX)

Table S2 **Internet Gaming Disorder 9 Criteria, Instructions and Reliability.**
(DOCX)
