# Supplementary material for: The Conceptualisation and Measurement of DSM-5 Internet Gaming Disorder: The Development of the IGD-20 Test
Source: PLoS One. 2014 Oct 14;9(10):e110137. doi: 10.1371/journal.pone.0110137 (PMC4196957; doi:10.1371/journal.pone.0110137)
Supplement: Table S1 — The Internet Gaming Disorder Test, Dimensionality and Instructions. (DOCX) [file pone.0110137.s001.docx]

**Table S1.** The Internet Gaming Disorder Test, Dimensionality and Instructions.

| **Internet Gaming Disorder Test (IGD Test)*** |
| --- |
| 1. I often lose sleep because of long gaming sessions. |
| 2R**. I never play games in order to feel better. |
| 3. I have significantly increased the amount of time I play games over last year. |
| 4. When I am not gaming I feel more irritable. |
| 5. I have lost interest in other hobbies because of my gaming. |
| 6. I would like to cut down my gaming time but it’s difficult to do. |
| 7. I usually think about my next gaming session when I am not playing. |
| 8. I play games to help me cope with any bad feelings I might have. |
| 9. I need to spend increasing amounts of time engaged in playing games. |
| 10. I feel sad if I am not able to play games. |
| 11. I have lied to my family members because the amount of gaming I do. |
| 12. I do not think I could stop gaming. |
| 13. I think gaming has become the most time consuming activity in my life. |
| 14. I play games to forget about whatever’s bothering me. |
| 15. I often think that a whole day is not enough to do everything I need to do in-game. |
| 16. I tend to get anxious if I can’t play games for any reason. |
| 17. I think my gaming has jeopardised the relationship with my partner. |
| 18. I often try to play games less but find I cannot. |
| 19R**. I know my main daily activity (i.e., occupation, education, homemaker, etc.) has not been negatively affected by my gaming. |
| 20. I believe my gaming is negatively impacting on important areas of my life. |
| **Dimensions** |
| Salience: 1, 7, 13 |
| Mood Modification: 2R, 8, 14 |
| Tolerance: 3, 9, 15 |
| Withdrawal Symptoms: 4, 10, 16 |
| Conflict: 5, 11, 17, 19R, 20 |
| Relapse: 6, 12, 18 |
|  |

*Instructions: These questions relate to your gaming activity during the past year (i.e., 12 months). By gaming activity we mean any gaming-related activity that was played on either a computer/laptop, gaming console and/or any other kind of device online and/or offline.

** Reversely score items.

*** Items answered in a 5-poin scale: 1 “strongly disagree”, 2 “disagree”, 3 “neither agree or disagree”, 4 “agree”, 5 “strongly agree”.

**** Suggested empirical cut-off for the test: 71 points.
