# Supplementary material for: The Conceptualisation and Measurement of DSM-5 Internet Gaming Disorder: The Development of the IGD-20 Test
Source: PLoS One. 2014 Oct 14;9(10):e110137. doi: 10.1371/journal.pone.0110137 (PMC4196957; doi:10.1371/journal.pone.0110137)
Supplement: Table S2 — Internet Gaming Disorder 9 Criteria, Instructions and Reliability. (DOCX) [file pone.0110137.s002.docx]

**Table S2.** Internet Gaming Disorder 9 Criteria, Instructions and Reliability.

| **Modified Internet Gaming Disorder 9 criteria (DSM-5) (APA, 2013)*** |
| --- |
|  |
| 1. Do you feel preoccupied with your gaming behaviour? (Some examples: Do you think about previous gaming activity or anticipate the next gaming session? Do you think gaming has become the dominant activity in your daily life?) |
| 2. Do you feel more irritability, anxiety or even sadness when you try to either reduce or stop your gaming activity? |
| 3. Do you feel the need to spend increasing amount of time engaged gaming in order to achieve satisfaction or pleasure? |
| 4. Do you systematically fail when trying to control or cease your gaming activity? |
| 5. Have you lost interests in previous hobbies and other entertainment activities as a result of your engagement with the game? |
| 6. Have you continued your gaming activity despite knowing it was causing problems between you and other people? |
| 7. Have you deceived any of your family members, therapists or others because the amount of your gaming activity? |
| 8. Do you play in order to temporarily escape or relieve a negative mood (e.g., helplessness, guilt, anxiety)? |
| 9. Have you jeopardised or lost an important relationship, job or an educational or career opportunity because of your gaming activity? |
|  |

*Instructions: These questions will ask you about your gaming activity during the past year (i.e., last 12 months). By gaming activity we understand any gaming-related activity that has been played either from a computer/laptop or from a gaming console or any other kind of device (e.g., mobile phone, tablet, etc.) both online and/or offline.

** Items answered in a 5-poin scale: 1 “never”, 2 “rarely”, 3 “sometimes”, 4 “often”, 5 “very often”.

*** Reliability (Cronbach’s Alpha) for the 9 criteria = .87.
